# Adaptive dosing of anticancer drugs in neonates: facilitating evidence-based dosing regimens

**DOI:** 10.1007/s00280-016-2975-0

**Published:** 2016-02-13

**Authors:** Gareth J. Veal, Julie Errington, Jairam Sastry, Julia Chisholm, Penelope Brock, Daniel Morgenstern, Kathy Pritchard-Jones, Tanzina Chowdhury

**Affiliations:** Northern Institute for Cancer Research, Paul O’Gorman Building, Medical School, Framlington Place, Newcastle University, Newcastle upon Tyne, NE2 4HH UK; School of Medicine, Glasgow University, Glasgow, G12 8QQ UK; Great Ormond Street Hospital, London, WC1N 3JH UK; Institute of Child Health, University College London, London, WC1N 1EH UK

**Keywords:** Neonates, Chemotherapy, Hepatoblastoma, Neuroblastoma, Wilms’ tumour, Cisplatin, Vincristine, Carboplatin, Etoposide, Therapeutic drug monitoring

## Abstract

**Purpose:**

Selection of the most appropriate chemotherapy dosing regimens for neonates treated within the first weeks of life represents a significant clinical dilemma. Due to a lack of information relating to the clinical pharmacology of anticancer drugs in these challenging patients, current dosing guidelines are based on limited scientific rationale. In the current study, we investigate the utilisation of therapeutic drug monitoring approaches in neonates with localised hepatoblastoma, Wilms’ tumour and stage 4S neuroblastoma, being treated with widely used anticancer drugs.

**Methods:**

Plasma concentrations of cisplatin, vincristine, etoposide and carboplatin were quantified in two neonates being treated within the first 3 weeks of life and in a 32-week preterm infant treated at a gestational age of 40 weeks. Therapeutic drug monitoring was carried out where appropriate, based on the pharmacokinetic data obtained in conjunction with clinical response and toxicity.

**Results:**

Treatment of a child aged 2 weeks with a recommended cisplatin dose reduction for weight to 1.8 mg/kg resulted in achievement of unbound cisplatin plasma concentrations of 0.01–0.08 µg/mL, markedly lower than exposures previously reported in infants and older children. A dose increase to 2.7 mg/kg was implemented, leading to the achievement of levels more in-line with those previously reported. This increased dose level was well tolerated over six courses of treatment, resulting in a good response to cisplatin monotherapy and the patient remains in remission at 3.5 years. In contrast, a 50 % vincristine dose reduction for weight in a 3-week-old neonate resulted in plasma concentrations comparable to levels observed in older children, leading to successful treatment and continued remission at 2 years. In a third patient, etoposide and carboplatin clearance values normalised to body weight were comparable to those reported in older children, resulting in comparatively lower exposures following reduced dosing.

**Conclusions:**

The current report provides unique data on the pharmacokinetics of several widely used anticancer drugs in neonates treated within the first few weeks of life. The provision of these data acts as a useful reference point to support future dosing decisions to be made by clinicians in the treatment of these challenging patients.

## Introduction

Selection of the most appropriate dosing regimen to administer to neonates within the first few weeks of life represents a significant clinical dilemma. Developmental physiological changes that occur following birth have the potential to significantly impact drug disposition and therefore the likely clinical outcome, in terms of observed response and toxicity [1, 2]. It is therefore a concern that there currently exists a lack of scientific rationale and standardisation for many well established drugs, in terms of the dosing regimens implemented for these very young infants, with many commonly utilised regimens largely defined for children between 3 and 12 months of age [3]. Markedly reduced dosing would appear to be a widely accepted approach in these patients, largely driven by concerns over an increased susceptibility to the side effects of treatment in developing neonates and/or the premise that drugs will be handled differently than in older children in terms of drug disposition. However, in the vast majority of cases there would appear to be limited scientific rationale on which to base these assumptions.

There currently exist relatively limited data concerning anticancer drug disposition in the neonatal patient population. While some excellent review papers in this area have previously been published, they highlight the dearth of information currently available from pharmacokinetic studies in infant patients per se and a complete lack of data generated from studies within the first few weeks of life for all but a handful of anticancer drugs [4, 5]. Where studies have been carried out, drug disposition has generally been shown to differ from that observed in older children and adults, most likely relating to the continuing development and maturation of renal and hepatic function in the neonate, which may significantly impact on drug metabolism and elimination [6, 7].

Previous studies carried out by our group have highlighted the clinical benefits of carrying out therapeutic drug monitoring (TDM) approaches with the anticancer drug carboplatin. These studies have included the treatment of preterm and newborn neonates, diagnosed with retinoblastoma and treated within the first few weeks of life [8, 9]. The generation of additional pharmacokinetic data in this patient population, for a wider array of anticancer drugs and tumour types, will facilitate the implementation of more appropriate evidence-based dosing regimens for the future treatment of neonates with cancer.

In the current study, we describe a series of case reports in neonates being treated with the anticancer drugs cisplatin, vincristine, etoposide and carboplatin for a range of tumour types within the first weeks of life. The marked interpatient variability previously observed for all four of these drugs in older children, combined with the known maturational changes in developing infants, provides a convincing case supporting the implementation of TDM studies with each course of therapy. Publication of these unique data, even in small patient numbers, will help to provide some guidance and rationale for the future treatment of neonates with these commonly used anticancer drugs.

## Materials and methods

### Patient treatment

Three neonates being treated with various anticancer drugs were studied at two clinical centres in the UK. The first patient (001) was born full term, diagnosed with localised hepatoblastoma and treated with cisplatin monotherapy at 2 weeks of age and a body weight (BW) of 3.0 kg. The patient had a creatinine level of 22 µmol/L (within the normal range for this age [10]), liver function tests as measured by AST and ALT values of 29 and 9 U/L, respectively, and an albumin level of 22 g/L (all within normal ranges based on age [11]). An elevated bilirubin level of 199 µmol/L was observed (predominantly unconjugated bilirubin associated with neonatal jaundice) in addition to a highly elevated LDH level of 1067 U/L. An initial cisplatin dose of 5.4 mg (1.8 mg/kg) was administered as a 24-h intravenous infusion on course 1 of treatment, and blood samples were collected for quantification of cisplatin plasma concentrations. Prior to course 2 of treatment, the patient had a creatinine level of 21 µmol/L, AST and ALT values of 39 and 13 U/L, respectively, and an albumin level of 25 g/L (all within normal ranges based on age [10, 11]). The elevated bilirubin level measured on course 1 of treatment had reduced to 44 µmol/L prior to cisplatin administration on course 2. The dose of cisplatin was increased to 8.3 mg (2.7 mg/kg) on course 2 of treatment, based on the plasma concentrations observed on course 1 and how the patient tolerated treatment, with blood samples for pharmacokinetic analysis again collected.

The second patient (002) was born full term and diagnosed with Wilms’ tumour (stage 1 intermediate risk) on day 2 of life. They received weekly vincristine following a primary nephrectomy, according to the protocol recommended dose of 1.5 mg/m^2^ vincristine reduced by 50 % due to the very young age of the patient (3 weeks of age, BW 3.3 kg). A dose of 0.16 mg (0.05 mg/kg, 0.75 mg/m^2^) was administered as a short intravenous infusion, with blood samples collected for quantification of vincristine plasma concentrations. The patient had a creatinine level of 46 µmol/L (within the normal range for this age [10]) at initiation of chemotherapy, normal liver function tests (ALT 17 U/L, ALP 147 U/L), a bilirubin level of 13 µmol/L and an albumin level of 29 g/L (within normal ranges for patient age [11]). Albumin levels were seen to progressively increase to from 29 to 34 g/L during treatment.

The third patient (003) received carboplatin, etoposide and vincristine for the treatment of stage 4S neuroblastoma with MYCN amplification. The patient had been born 8 weeks premature at a gestational age of 32 weeks and was suffering from anuria when treated at age 8 weeks (gestational age 40 weeks), with a body weight of 2.5 kg. At the time of treatment, the patient had a creatinine level of 43 µmol/L (within the normal range for this age [10]), a relatively low albumin level (21 g/L), elevated ALT (97 U/L) and elevated bilirubin (54 µmol/L), consistent with acute liver injury secondary to tumour and hepatic artery ligation. Blood samples were collected for the analysis of carboplatin and etoposide plasma concentrations. Carboplatin was administered at a dose of 10 mg (4 mg/kg) as a 1-h intravenous infusion on days 1, 2 and 3 of treatment. Etoposide was administered as a 4-h intravenous infusion at a dose of 8 mg (3.2 mg/kg) on day 1 and at a dose of 12.5 mg (5 mg/kg) on days 3 and 4 of treatment, with vincristine administered as a short intravenous infusion at a dose of 0.28 mg (0.11 mg/kg) on day 4 only. The patient underwent continuous veno-venous haemofiltration (CVVH) before and after chemotherapy due to oligo-anuria.

Patient characteristics are provided alongside details of individual patient treatment in Table 1. Ethical approval was not required for this approach to treatment, providing information on drug levels as a clinical request by the treating centre to be used alongside tolerability data to guide the selection of dosing regimens over multiple courses of treatment. TDM in this setting was carried out at the request of the treating clinician.

**Table 1:** Patient characteristics and treatment details

Patient	Tumour type	Age at time of treatment	BW (kg)	Initial treatment and dosing regimens	Number of treatment courses	Additional information
001	Hepatoblastoma	2 weeks	3.0	Single-agent cisplatin: 1.8 mg/kg on course 1; 2.7 mg/kg on course 2	6	Plasma levels on course 1 used to guide dose increase on course 2. Each course consisted of a single dose of cisplatin with 2 weeks between courses
002	Wilms’ tumour	3 weeks	3.3	Single-agent vincristine at 50 % reduced dose level (0.16 mg; 0.75 mg/m^2^)	10	Dose maintained at 50 % reduced dose level due to achievement of vincristine plasma concentrations equivalent to those observed in older children
003	Neuroblastoma (stage 4S)	8 weeks (gestational age: 40 weeks)	2.5	Carboplatin: 10 mg on days 1,2 and 3; Etoposide: 8 mg on day 2, 12.5 mg on days 3/4; Vincristine: 0.28 mg on day 4	1	Blood samples collected on days 1, 2 and 3 of treatment. Patient underwent continuous veno-venous haemofiltration before and after chemotherapy due to oligo-anuria

### Blood sampling and analysis

For patient 001, blood samples (1 mL) for pharmacokinetic analysis were obtained from a central line prior to cisplatin infusion, 6 h after the start of infusion, at 24 h (end of infusion) and at 8 h following the end of infusion. Plasma was separated from whole blood samples by centrifugation (1200 g, 4 °C, 10 min), and 0.5 mL was then removed and placed in an Amicon Centrifree micropartition unit with a 30,000 MW cut-off (Millipore, Edinburgh, UK). This sample was centrifuged (1500 g, 4 °C, 15 min) to obtain plasma ultrafiltrate for determination of free cisplatin levels. For patient 002, blood samples (2 mL) were collected at 30 min, 2 h, 6 h and 24 h following a short bolus infusion of vincristine, and plasma was immediately separated by centrifugation (1200 g, 4 °C, 10 min). For patient 003, blood samples were obtained before carboplatin infusion, 30 min after the start of infusion, at 1 h (end of infusion) and at 1 and 2 h after the end of infusion on days 1 and 2 of treatment. Plasma was separated from whole blood samples by centrifugation (1200 g, 4 °C, 10 min), and 0.5 mL was then removed for preparation of plasma ultrafiltrate as described above, for determination of free carboplatin levels. For analysis of etoposide levels in this patient, blood samples were collected at 2 h after the start of infusion, at 4 h (end infusion) and at 2 h after the end of infusion on day 1 of treatment, and plasma was immediately separated by centrifugation (1200 g, 4 °C, 10 min). All samples for quantification of drug levels were stored at −20 °C prior to analysis.

Samples were sent by overnight courier, on dry ice and in an insulated container, to the Northern Institute for Cancer Research, Newcastle University. Cisplatin and carboplatin pharmacokinetic analyses were carried out by flameless atomic absorption spectrophotometry (AAS) using a Perkin–Elmer AAnalyst 600 graphite furnace spectrometer (Perkin–Elmer Ltd, Beaconsfield, UK). Total and free (unbound) platinum levels were determined in plasma and plasma ultrafiltrate samples, respectively, as described previously [12, 13]. All samples were analysed in duplicate, and values expressed as the average of these measurements. Duplicate values were within 15 % of each other in all cases. Intra- and inter-assay coefficients of variation for a quality assurance sample had to be <10 % for an assay to be valid. The limit of detection for the AAS assay was 0.10 µg/mL. Quantification of vincristine levels in plasma samples was carried out using a validated liquid chromatography–mass spectrometry (LC/MS) assay, with a lower limit of quantification (LLOQ) of 0.50 ng/mL as previously described [14]. Etoposide plasma concentrations were determined using an API 2000 LC/MS assay, with a standard curve of 0.20–10.0 µg/mL as previously described [15]. Intra-assay coefficients of variation were <10 % in all cases.

### Statistical analysis

Pharmacokinetic parameters for cisplatin, vincristine and etoposide were calculated by non-compartmental analysis using the WinNonlin software package. Carboplatin clearance (Cl) and AUC were determined by Bayesian analysis using a two-compartment model as described previously [13, 16].

## Results

### Drug treatment and dose adjustment

**Fig. 1:**
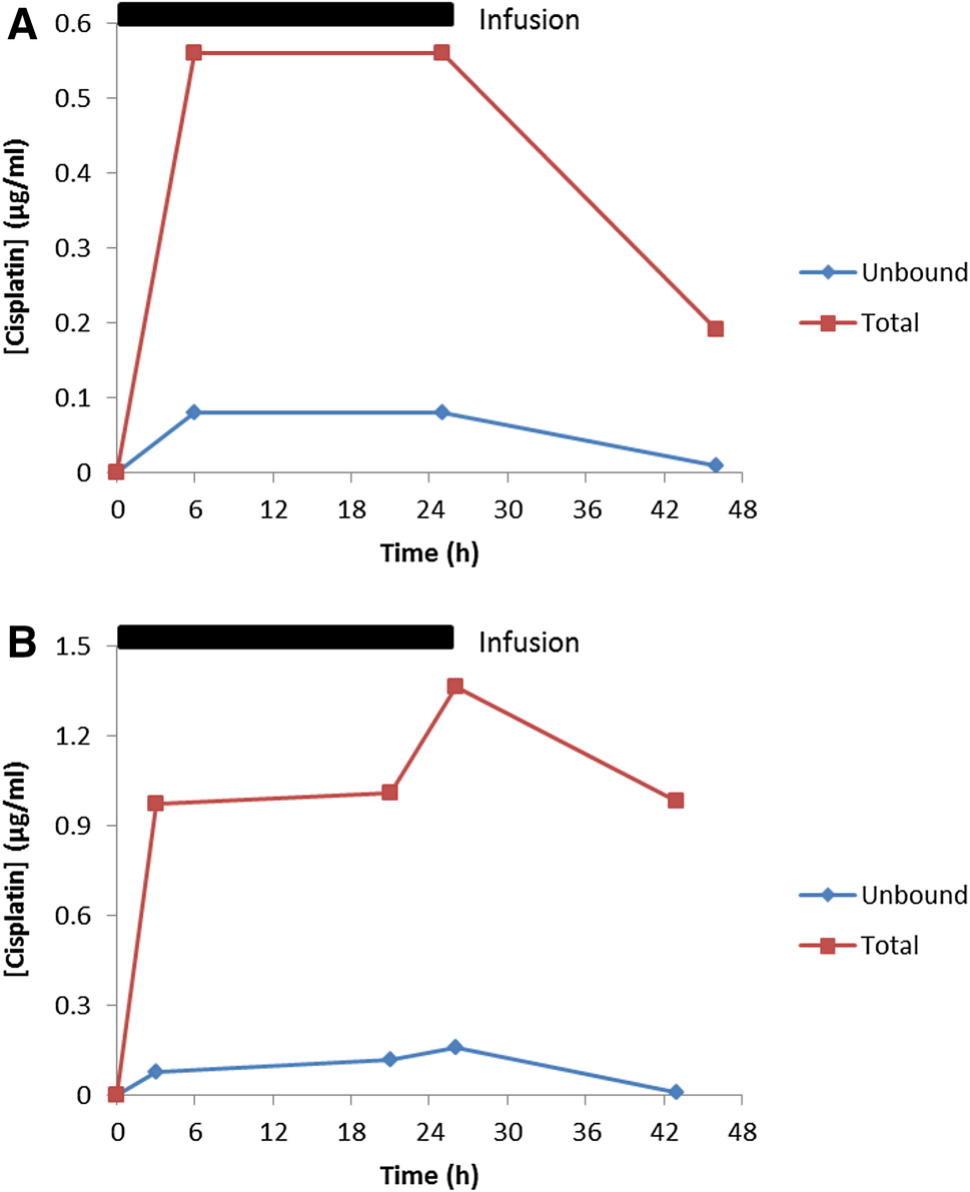
Plasma concentrations of unbound and total cisplatin measured in patient 001 following treatment with cisplatin administered as a 24-h intravenous infusion at a dose of 1.8 mg/kg at 2 weeks of age on course 1 (**a**) and a dose of 2.7 mg/kg at 4 weeks of age on course 2 (**b**)

**Fig. 2:**
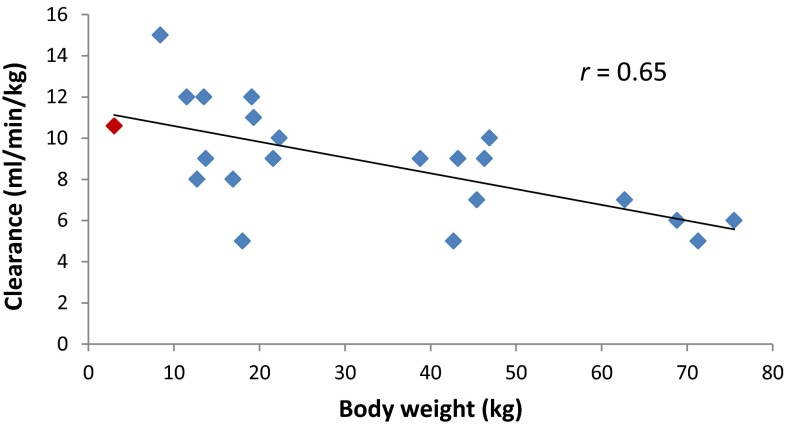
Relationship between patient body weight and unbound cisplatin clearance normalised to body weight in a paediatric patient population (data from a previously published study [17] with data included from patient 001 shown in *red*)

Patient 001 was treated with cisplatin at 2 weeks of age (BW 3.0 kg) at an initial dose of 1.8 mg/kg administered as a 24-h intravenous infusion. Concentrations of unbound cisplatin measured in plasma ultrafiltrate, determined over a 48-h period following the start of administration, ranged from 0.01 to 0.08 µg/mL, with corresponding total cisplatin concentrations in plasma ranging from 0.19 to 0.56 µg/mL (Fig. 1a). These levels are approximately fivefold lower than concentrations previously reported in childhood cancer patients receiving cisplatin for the treatment of hepatoblastoma and other tumour types [12]. Based on the low plasma concentrations observed on course 1 of treatment, the dose of cisplatin was increased by 50 % to a dose of 2.7 mg/kg on course 2 (at 4 weeks of age) and levels again quantified. Following this dose increase, concentrations of unbound cisplatin ranged from 0.12 to 0.16 µg/mL, with corresponding total cisplatin concentrations in plasma ranging from 0.97 to 1.36 µg/mL (Fig. 1b). Table 2 shows the key pharmacokinetic parameters determined following doses of 1.8 and 2.7 mg/kg in this patient. Unbound cisplatin Cl values of 10.6 and 10.1 mL/min/kg were observed on course 1 and 2 of treatment, following doses of 1.8 and 2.7 mg/kg, respectively, with corresponding AUC_0–∞_ values of 168 and 266 µg/mL min. These data compare to unbound cisplatin Cl values of 3–15 mL/min/kg and AUC values of 300–810 µg/mL min previously reported in children aged 0.5–19.3 years of age, following a dose of 100 mg/m^2^ (approximately 3–5 mg/kg) cisplatin, similarly administered as a 24-h intravenous infusion [17]. Inclusion of the data obtained in the current study with these previously published data indicates an apparent relationship between patient BW and normalised cisplatin clearance, with a trend towards proportionally higher clearance values in smaller patients (Fig. 2). Unbound cisplatin *t*_1/2_ values observed in the current study were 7.0 and 4.3 h on courses 1 and 2 of treatment, respectively, whereas total cisplatin *t*_1/2_ values increased from 13.5 h on course 1 to 36.0 h on course 2 of treatment.

**Table 2:** Pharmacokinetic parameters for unbound and total cisplatin following administration of 1.8 mg/kg (course 1) and 2.7 mg/kg (course 2) cisplatin as a 24-h intravenous infusion to a 2-week-old neonate

Cisplatin dose (mg/kg)	Total cisplatin			Unbound cisplatin		
	Cmax (µg/mL)	AUC_0–∞_ (µg/mL min)	Cl (mL/min/kg)	Cmax (µg/mL)	AUC_0–∞_ (µg/mL min)	Cl (mL/min/kg)
1.8	0.56	1433	1.24	0.08	168	10.6
2.7	1.36	5756	0.47	0.16	266	10.1

**Fig. 3:**
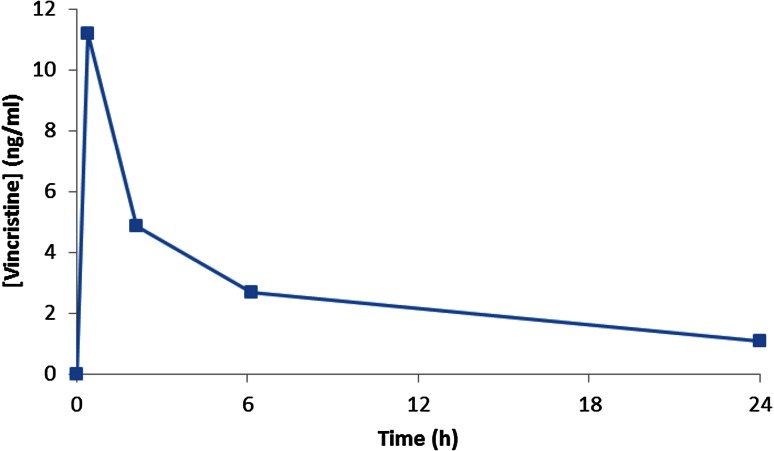
Plasma concentrations of vincristine measured in patient 002 following short intravenous administration of a single dose of 0.75 mg/m^2^ (0.05 mg/kg)

Patient 002 received vincristine at 3 weeks of age (BW 3.3 kg), as a short intravenous infusion at a 50 % reduced dose level of 0.75 mg/m^2^, equivalent to 0.05 mg/kg. Vincristine concentrations ranged from 11.2 ng/mL at 30 min post-infusion to 1.1 ng/mL at 24 h (Fig. 3), comparable to levels reported in older children (3–9 years of age) with Wilms’ tumour, following the recommended full dose of 1.5 mg/m^2^ for children weighing >12 kg (range of 5.0–12.4 ng/mL at 30 min and 1.1–2.3 ng/mL at 24 h) [14]. Vincristine pharmacokinetics calculated by non-compartmental analysis included a Cl of 29.8 mL/min (142 mL/min/m^2^) and an AUC_0–24h_ of 4066 ng/mL min, as compared to previously reported Cl values of 120–897 mL/min (169–825 mL/min/m^2^) and AUC_0–24h_ values of 1591–8310 ng/mL min [14]. Table 3 shows the key pharmacokinetic parameters determined for vincristine in this patient.

**Table 3:** Pharmacokinetic parameters for vincristine following administration of 0.75 mg/m^2^ (0.05 mg/kg) vincristine as a short intravenous infusion in a 3-week-old neonate

Dose (mg/kg)	Dose (mg)	Cl (mL/min)	Cl (mL/min/m^2^)	AUC_0–24h_ (ng/mL min)	*t*_1/2_ (h)	Vz (L)
0.05	0.16	29.8	142	4066	13.8	35.5

Patient 003 received carboplatin, etoposide and vincristine for the treatment of stage 4S neuroblastoma with MYCN amplification, at a gestational age of 40 weeks (BW 2.5 kg). Plasma concentrations of carboplatin and etoposide were quantified on day 1, with carboplatin levels again analysed on day 2 of treatment. Concentrations of unbound carboplatin following a dose of 4 mg/kg/day peaked at 7.3 and 6.6 µg/mL on days 1 and 2 of treatment, respectively, with corresponding AUC values of 2.3 and 1.1 mg/mL min observed. Carboplatin Cl values of 4.4 mL/min (1.8 mL/min/kg) on day 1 of treatment and 9.4 mL/min (3.8 mL/min/kg) on day 2 were calculated. These values compare with previously published data from infant neuroblastoma patients aged between 2 and 14 months of age, which showed a range of carboplatin Cl values of 12.8–33.6 mL/min (2.1–3.5 mL/min/kg) and achievement of AUC values of 1.4–3.1 mg/mL min following a carboplatin dose of 6.6 mg/kg recommended for children <12 kg [15].

Etoposide concentrations determined in patient 003 following a dose of 3.2 mg/kg peaked at a plasma concentration of 6.1 µg/mL at the end of a 4-h intravenous infusion. Table 4 shows the key pharmacokinetic parameters for etoposide in this patient studied at a gestational age of 40 weeks. Etoposide Cl was 1.95 mL/min (0.78 mL/min/kg), with an observed AUC value of 4.1 mg/mL min. These values compare to reported AUC values ranging from 3.4 to 11.0 mg/mL min and Cl values of 3.2–13.0 mL/min (0.46–1.45 mL/min/kg) in a previous study investigating etoposide pharmacokinetics in infant patients aged 2–14 months of age, following an etoposide dose of 5 mg/kg/day administered as a 2-h infusion.

**Table 4:** Pharmacokinetic parameters for etoposide following administration of 3.2 mg/kg etoposide as a 4-h intravenous infusion in a preterm infant at an age of 8 weeks (gestational age of 40 weeks)

Dose (mg/kg)	Dose (mg)	Cl (mL/min)	Cl (mL/min/kg)	AUC_0–∞_ (mg/mL min)	*t*_1/2_ (h)	Vz (L)
3.2	8	1.95	0.78	4.1	5.8	1.0

### Treatment response and toxicity

Patient 001 received a total of six courses of cisplatin, with the dose increased on course 2 based on plasma concentrations observed on course 1. The increased dose level of 2.7 mg/kg was then maintained for courses 3–6 of treatment with no grade 3/4 toxicities reported. The patient responded well to cisplatin monotherapy, allowing complete surgical resection, and is now in complete remission 3.5 years from the end of treatment. Patient 002 received weekly vincristine for 10 weeks post-nephrectomy, following diagnosis of Wilms’ tumour on day 2 of life. No toxicities were observed following treatment and the patient remains in remission at 2 years of age. Patient 003 was suffering from anuria at the time of treatment and died 6 days after the start of chemotherapy with hepatomegaly, coagulopathy, haemodynamic compromise and renal failure related to disease progression.

## Discussion

Administration of the most appropriate doses of anticancer drugs to neonates within the first few weeks of life represents a significant clinical challenge. Dose adjustments recommended in clinical trial and treatment protocols are generally extrapolated from experience in dosing older infants. While it may be unrealistic to design large prospective clinical trials to generate data in large numbers of children within this defined population, it is essential that clinical pharmacology data generated are published to help guide future patient treatment. This is particularly important bearing in mind the potential impact of ontogeny on expression of drug transporters and hence drug disposition, which is likely to be most relevant to neonates and preterm infants [18]. Without the publication of case reports and minimal datasets on small numbers of patients, clinicians will continue to struggle with the same concerns and uncertainties when treating preterm infants and neonates with cancer, and the actual impact of ontogeny on drug disposition will be difficult to assess. In order to improve the knowledge base in this area and have a real impact on clinical treatment, international collaboration and sharing of data will be required, ideally in the form of a single accessible database.

In the current study, we report on pharmacokinetic data for the anticancer drugs cisplatin, vincristine, etoposide and carboplatin, generated in two neonates being treated within the first 3 weeks of life and in a 32-week preterm infant treated at a gestational age of 40 weeks. It is currently common practice to dose infant patients (less than 1 year of age) based on BW, alongside additional dose reductions, such as a 50 % dose reduction for children <6 months of age [5, 19]. However, this may not necessarily be the most clinically advantageous approach [19, 20]. While it is essential that developing neonates are not subject to unnecessary side effects associated with their treatment, it is equally important that clinically meaningful exposures of drugs are achieved, in order for them to exhibit appropriate anti-tumour activity.

Treatment of a neonate diagnosed with hepatoblastoma at 2 weeks of age provided an opportunity to generate pharmacokinetic data on cisplatin in this very young child, with a view to guiding therapy on course 2 of treatment in addition to providing information that may be useful for guiding future patient treatment in similar clinical situations. As reported by van den Berg et al. [5], dose recommendations for cisplatin are difficult to provide due to the lack of pharmacokinetic data published in infants. Following administration of a recommended dose of 1.8 mg/kg on course 1 of treatment in this patient, cisplatin levels were markedly lower than concentrations previously reported in the literature for children being treated for hepatoblastoma and other tumour types [12]. A 50 % dose increase to a dose level of 2.7 mg/kg was therefore implemented on course 2 of treatment, resulting in the achievement of plasma concentrations comparable to those previously reported in older children. As has previously been indicated for carboplatin [15], it may well be the case that cisplatin clearance values normalised for BW are proportionally higher in neonates and infants than in older children. Such differences may be related to a reduction in protein binding in neonates, leading to a relative increase in renal and/or metabolic drug clearance, may be linked to differences in organ size relative to total body weight, or may reflect differences in ratios of fat, protein and intracellular water in this very young patient population. If this is the case, then these data would clearly not support the reduced dosing levels commonly proposed for these very young patients. While unbound cisplatin Cl was comparable between courses 1 and 2 of treatment, a marked difference in total cisplatin Cl was observed between courses (1.24 mL/min/kg on course 1 vs. 0.47 mL/min/kg on course 2), leading to a threefold increase in total cisplatin AUC. This could not be explained by a difference in albumin levels between course 1 and 2 of treatment (22 vs. 25 g/L, respectively), although bilirubin levels were markedly elevated prior to treatment on course 1 but not course 2. The differences in total cisplatin pharmacokinetics observed between two courses of treatment separated by only 2 weeks may indicate that constant monitoring of drug levels across sequential courses may be prudent for future neonate cancer patients.

Although it cannot be ruled out that the patient may have responded to treatment at the lower exposures observed, from a pharmacological perspective, such low cisplatin plasma concentrations would be a cause for concern and it is encouraging that the higher dose was well tolerated in this case. For the patient studied here, the increased dose of 2.7 mg/kg cisplatin was continued for courses 2–6 of treatment, with minimal toxicity observed, and the patient now remains in complete remission over 3 years following the end of treatment. This kind of TDM approach, utilising pharmacokinetic parameters to guide treatment, has previously been suggested for treating young patients with cisplatin due to the uncertainties of currently available dosing guidelines [21].

In contrast to the results obtained following cisplatin treatment in a neonate, implementation of a 50 % dose reduction for a 3-week-old Wilms’ tumour patient being treated with vincristine resulted in achievement of a drug exposure comparable to values previously reported in older children [14]. The generation of these data in a TDM setting provided the clinical team with the confidence to maintain this dose level for 10 weeks of treatment, leading to ongoing complete remission in the absence of significant toxicity. These findings are supported by data from a previous study by Crom et al. [22], including pharmacokinetic data obtained from twins aged 2 months, indicating that dosing should be based on BW as opposed to BSA in infant patients. In this neonate setting, a 50 % reduction in dose for vincristine would appear to be appropriate.

The final patient studied was a 32-week preterm infant diagnosed with stage 4S neuroblastoma and MYCN amplification, being treated at 8 weeks of age (gestational age of 40 weeks). Although this patient died only 6 days after the start of treatment, due to renal failure associated with disease progression, the pharmacokinetic data obtained again provide useful information in a rare and challenging clinical situation. Although carboplatin clearance values varied over the 2 days of pharmacokinetic sampling, they were comparable to those reported in previously studied infant neuroblastoma patients, resulting in the achievement of lower AUC values due to the lower dose of 4 mg/kg/day implemented in this patient, as compared to a recommended dose of 6.6 mg/kg [15]. Similarly, for etoposide, the dose utilised (3.2 mg/kg) was lower than a standard dose of 5 mg/kg recommended in infant neuroblastoma patients [15, 23]. The etoposide clearance value observed in the current study was within the range previously reported in infants (0.46–1.45 mL/min/kg) and older children [15, 24], and the observed AUC therefore relatively low in comparison with this patient group, due to the lower dose administered. No conclusions can be drawn in relation to the pharmacokinetics of carboplatin and etoposide and clinical response or toxicity in this patient, as death due to disease progression occurred only days after treatment. In addition, the patient was receiving continuous veno-venous haemofiltration (CVVH) before and after chemotherapy due to oligo-anuria and the impact of this on the pharmacokinetic parameters observed is unclear. However, the data generated would suggest that higher doses of carboplatin and etoposide may be beneficial in infant patients, assuming that they are appropriately tolerated. Indeed, this may be particularly relevant in the case of neonates being treated for stage 4S neuroblastoma, where outcome is extremely favourable if initial disease burden causing life-threatening organ compromise can be overcome. In such a clinical situation, with a short window of opportunity to control rapidly growing abdominal disease in a high-risk situation, administration of the most appropriate and effective chemotherapy dosing regimen is likely to be critical.

The current report provides novel data on the pharmacokinetics of several widely used anticancer drugs in neonates treated within the first few weeks of life and provides a comparison with data generated in older infants and children where data are available. The provision of these clinical pharmacology data in a challenging patient population acts as a useful reference point to support future dosing decisions to be made by clinicians in the treatment of preterm infants and neonates within the first few weeks of life. The generation and publication of datasets such as these provide useful clinical direction and are essential if we are to develop meaningful dosing guidance for commonly used anticancer drugs in this patient population. Such data will allow decisions to be made as to the most appropriate dosing regimens to be utilised and identify which agents may require dose optimisation through TDM approaches on single or multiple courses of treatment.
